# Variation at the capsule locus, *cps*, of mistyped and non-typable *Streptococcus pneumoniae* isolates

**DOI:** 10.1099/mic.0.056580-0

**Published:** 2012-06

**Authors:** S. J. Salter, J. Hinds, K. A. Gould, L. Lambertsen, W. P. Hanage, M. Antonio, P. Turner, P. W. M. Hermans, H. J. Bootsma, K. L. O'Brien, S. D. Bentley

**Affiliations:** 1 Wellcome Trust Sanger Institute, Hinxton, UK; 2 St George’s, University of London, UK; 3 Statens Serum Institut, Copenhagen, Denmark; 4 Harvard School of Public Health, Boston, MA, USA; 5 Medical Research Council Laboratories, Fajara, The Gambia; 6 Shoklo Malaria Research Unit, Mae Sot, Thailand; 7 Centre for Tropical Medicine, University of Oxford, UK; 8 Radboud University Nijmegen Medical Centre, Nijmegen, The Netherlands; 9 Johns Hopkins Bloomberg School of Public Health, Baltimore, MD, USA

## Abstract

The capsule polysaccharide locus (*cps*) is the site of the capsule biosynthesis gene cluster in encapsulated *Streptococcus pneumoniae*. A set of pneumococcal samples and non-pneumococcal streptococci from Denmark, the Gambia, the Netherlands, Thailand, the UK and the USA were sequenced at the *cps* locus to elucidate serologically mistyped or non-typable isolates. We identified a novel serotype 33B/33C mosaic capsule cluster and previously unseen serotype 22F capsule genes, disrupted and deleted *cps* clusters, the presence of *aliB* and *nspA* genes that are unrelated to capsule production, and similar genes in the non-pneumococcal samples. These data provide greater understanding of diversity at a locus which is crucial to the antigenic diversity of the pathogen and current vaccine strategies.

## Introduction

*Streptococcus pneumoniae* is a widespread nasopharyngeal commensal and pathogen of humans, causing a range of conditions, including otitis media, sinusitis, pneumonia, septicaemia and meningitis, and is usually associated with disease in infants, the elderly and immunocompromised individuals. In the year 2000, an estimated 14.5 million cases of severe pneumococcal disease occurred worldwide in children aged under 5 years, causing approximately 11 % of all deaths in that age group (O’Brien *et al.*, 2009). The polysaccharide capsule, which has a variable structure divided into more than 90 serotypes, is the major known virulence factor, being important for survival in the blood and strongly associated with antiphagocytic activity (Kim *et al.*, 1999). The capsule induces protective antibodies, and is the basis for the 23-valent polysaccharide and 7-valent conjugate vaccines (pneumococcal conjugate vaccines; PCVs) that are licensed in over 70 countries, as well as the recently licensed 10- and 13-valent conjugate vaccines.

Some serotypes have been found to be more likely to occur in cases of invasive disease, relative to exposure through carriage (Brueggemann *et al.*, 2004; Hanage *et al.*, 2005). Before the use of the 7-valent conjugate vaccine in children, serotype 14 was the most common cause of invasive pneumococcal disease (IPD) globally (Johnson *et al.*, 2010). There is some evidence that certain capsule structures better enable survival in carriage and infection (Melin *et al.*, 2010; Weinberger *et al.*, 2009), although disease outcome can be independent of serotype in capsular switch experiments (Mizrachi Nebenzahl *et al.*, 2004). Since the introduction of the 7-valent vaccine among infants, cases of IPD attributable to vaccine serotypes have reduced, but other types persist in causing disease such as serotype 1 across Europe, Asia, Latin America and Africa (Kirkham *et al.*, 2006), or multidrug-resistant 19A in Spain (Muñoz-Almagro *et al.*, 2009), Israel (Dagan *et al.*, 2009), across Asia (Shin *et al.*, 2011) and in the USA (Beall *et al.*, 2011; Moore *et al.*, 2008).

In all but two serotypes, biosynthesis of the capsule is mediated by the Wzx/Wzy-dependent pathway encoded by genes at the *cps* (capsular polysaccharide synthesis) locus. The *cps* locus is located between the glucan 1,6-α-glucosidase gene *dexB* and the oligopeptide ABC transporter gene *aliA*, which are not involved in capsule synthesis. The Wzx/Wzy-dependent pathway involves several transferases that create a polysaccharide subunit which is polymerized and translocated across the membrane (Bentley *et al.*, 2006). Each serotype possesses a unique combination of *cps* genes or alleles. The alternative synthase pathway is found in two serotypes, 3 and 37. A single synthase gene is responsible for the production of these capsule types (Llull *et al.*, 1999; Paton & Morona, 2007).

Changes at the *cps* locus can affect capsule expression by several mechanisms. Slipped-strand mispairing causes a gene truncation, which is the root of the difference between serotypes 15B and 15C (van Selm *et al.*, 2003). The phenomenon of serotype switching refers to cases of recombination leading to the replacement of either a part or the entirety of the *cps* locus with the homologous region from a strain of another serotype. Serotype switching has been observed many times in nature (Croucher *et al.*, 2011) and demonstrated in the laboratory (Weinberger *et al.*, 2009). These variations have implications for serotype-specific vaccines, and several studies have shown switched clones arising in vaccinated populations (Ansaldi *et al.*, 2011; Brueggemann *et al.*, 2007; Temime *et al.*, 2008). As well as variation in capsule production during infection (Hammerschmidt *et al.*, 2005), spontaneous loss of capsule has been observed *in vitro*, where a single culture may sequentially lose and regain capsule production (Waite *et al.*, 2003), and may therefore be inconsistently reactive to typing sera.

Pneumococci designated non-typable (NT) may possess a capsule for which there are no typing antisera, they may produce the capsule erratically, or they may be non-encapsulated. Non-typable pneumococci are widely found in carriage studies and non-invasive disease episodes (Marsh *et al.*, 2010) but rarely in IPD. NT *S. pneumoniae* are poorly characterized compared with encapsulated strains, despite their common occurrence and potential for acting as a reservoir of genetic variety in the nasopharynx. The objective of this study was to investigate the *cps* gene content of pneumococci which are not serologically typable or which were shown by microarray analysis to possess non-standard capsular gene clusters. Isolates referred from a molecular typing array included those with non-standard combinations of identifiable *cps* genes, suspected deletions or novel genes, and other streptococci which appeared to possess pneumococcal-like genes.

## Methods

### 

#### Isolates.

Fifty-eight isolates, described in Tables 1 and S1, were obtained from Denmark (two *S. pneumoniae*, three non-pneumococcus), the Gambia (five *S. pneumoniae*, three non-pneumococcus), the Netherlands (nine *S. pneumoniae*, one non-pneumococcus), Thailand (18 *S. pneumoniae*, one non-pneumococcus), the UK (three *S. pneumoniae*, three non-pneumococcus) and the USA (10 *S. pneumoniae*). All isolates had been examined by capsular reaction test at the time of isolation, and DNA extracts were then analysed on the BµG@S SP-CPS v1.3.0 molecular serotyping array (Brugger *et al.*, 2010; Turner *et al.*, 2011) using standard Agilent 8×15K format array comparative genomic hybridization (array CGH) enzymic labelling and hybridization protocols. Fourteen pneumococcal isolates had no identifiable *cps* genes and no positive serological result, 30 had an incomplete list of genes and no serotype, three possessed genes that differed from their serological result, and 11 non-pneumococcal streptococci had pneumococcal *cps* genes.

**Table 1.** Summary of isolates

Sample name [GenBank accession no.]	Species	Country of origin	Serotype*	Non-typable group
557B [HE651321]	*S. pneumoniae*	Denmark	33C	Serotype and microarray results differ
L2008-01622 [HE651300]	*S. pneumoniae*	USA	22F	
1772-40b [HE651318]	*S. pneumoniae*	Denmark	22F	
2489-06 [HE651319]	*S. pneumoniae*	USA	Non-encapsulated	Group NT1: *cps* deletion
GM90852 [HE651312]	*S. pneumoniae*	The Gambia	Non-encapsulated	
GM108225 [HE651315]	*S. pneumoniae*	The Gambia	Non-encapsulated	
L2008-01621 [HE651299]	*S. pneumoniae*	USA	Non-encapsulated	
L2008-01629 [HE651301]	*S. pneumoniae*	USA	Non-encapsulated	
L2008-01630 [HE651302]	*S. pneumoniae*	USA	Non-encapsulated	
L2008-01636 [HE651303]	*S. pneumoniae*	USA	Non-encapsulated	
GM96650 [HE651314]	*S. pneumoniae*	The Gambia	Non-encapsulated	
07B00725 [HE651278]	*S. pneumoniae*	Thailand	Non-encapsulated	Group NT2: putative surface protein NspA
07B00751 [HE651279]	*S. pneumoniae*	Thailand	Non-encapsulated	
07B00782 [HE651280]	*S. pneumoniae*	Thailand	Non-encapsulated	
07B00890 [HE651283]	*S. pneumoniae*	Thailand	Non-encapsulated	
07B00933 [HE651286]	*S. pneumoniae*	Thailand	Non-encapsulated	
08B01575 [HE651297]	*S. pneumoniae*	Thailand	Non-encapsulated	
08B00936 [HE651287]	*S. pneumoniae*	Thailand	Non-encapsulated	
08B01425 [HE651289]	*S. pneumoniae*	Thailand	Non-encapsulated	
RUNMC819 [HE651281]	*S. pneumoniae*	The Netherlands	Non-encapsulated	
07B00830 [HE651282]	*S. pneumoniae*	Thailand	Non-encapsulated	
08B00930 [HE651285]	*S. pneumoniae*	Thailand	Non-encapsulated	
RUNMC2437 [HE651308]	*S. pneumoniae*	The Netherlands	Non-encapsulated	
0900-07 [HE651316]	*S. pneumoniae*	USA	Non-encapsulated	Group NT3: *aliB* genes
3039 [HE651275]	*S. pneumoniae*	UK	Non-encapsulated	
07-047 [HE651276]	*S. pneumoniae*	UK	Non-encapsulated	
RUNMC1664 [HE651304]	*S. pneumoniae*	The Netherlands	Non-encapsulated	
RUNMC1988 [HE651307]	*S. pneumoniae*	The Netherlands	Non-encapsulated	
RUNMC3306 [HE651310]	*S. pneumoniae*	The Netherlands	Non-encapsulated	
2566-06 [HE651320]	*S. pneumoniae*	USA	Non-encapsulated	
RUNMC1437 [HE651290]	*S. pneumoniae*	The Netherlands	Non-encapsulated	
RUNMC2945 [HE651309]	*S. pneumoniae*	The Netherlands	Non-encapsulated	
625 [HE651277]	*S. pneumoniae*	UK	Non-encapsulated	
RUNMC1897 [HE651306]	*S. pneumoniae*	The Netherlands	Non-encapsulated	
L2008-01618 [HE651298]	*S. pneumoniae*	USA	Non-encapsulated	
1878-08 [HE651317]	*S. pneumoniae*	USA	Non-encapsulated	
08B00915 [HE651284]	*S. pneumoniae*	Thailand	Non-encapsulated	
07B00945 [HE651288]	*S. pneumoniae*	Thailand	Non-encapsulated	
08B01481 [HE651292]	*S. pneumoniae*	Thailand	Non-encapsulated	
08B01482 [HE651293]	*S. pneumoniae*	Thailand	Non-encapsulated	
08B01463 [HE651291]	*S. pneumoniae*	Thailand	Non-encapsulated	
08B01483 [HE651294]	*S. pneumoniae*	Thailand	Non-encapsulated	
08B01531 [HE651296]	*S. pneumoniae*	Thailand	Non-encapsulated	
RUNMC1739 [HE651305]	*S. pneumoniae*	The Netherlands	Non-encapsulated	
08B01484 [HE651295]	*S. pneumoniae*	Thailand	Non-encapsulated	
GM15912 [HE651311]	*S. pneumoniae*	The Gambia	Non-encapsulated	
GM90967 [HE651313]	*S. pneumoniae*	The Gambia	Non-encapsulated	
IOPR 1791 [HE651264]	*S. pseudopneumoniae*	UK	Non-encapsulated	Non-pneumococcal streptococci
IOPR 5427 [HE651265]	*S. pseudopneumoniae*	UK	Non-encapsulated	
VS10 [HE651271]	*S. mitis*	UK	Non-encapsulated	
07B00902 [HE651266]	*S. pseudopneumoniae*	Thailand	Non-encapsulated	
RUNMC2031 [HE651272]	*S. mitis*	The Netherlands	Non-encapsulated	
GM56393 [HE651267]	*S. pseudopneumoniae*	The Gambia	Non-encapsulated	
GM66782 [HE651268]	*S. pseudopneumoniae*	The Gambia	Non-encapsulated	
GM73924 [HE651269]	Unidentified (*S. pneumoniae* or *S. pseudopneumoniae*)	The Gambia	Non-encapsulated	
1071-01 [HE651273]	*S. mitis*	Denmark	Non-encapsulated	
1298-02 [HE651274]	*S. mitis*	Denmark	Non-encapsulated	
958-02 [HE651270]	*S. pseudopneumoniae*	Denmark	Non-encapsulated	

* Serotype according to Quellung reaction.

#### Species identification.

Strains used in this study were identified at the time of isolation as *S. pneumoniae* or other streptococcal species by standard microbiological methods such as optochin sensitivity and bile solubility. The assigned species were confirmed by array CGH analysis of the *S. pneumoniae* genome backbone component of the molecular serotyping array, then further verified by sequencing of 16S rRNA genes and manual analysis of the V2, V4 and V5 hypervariable regions compared with a reference set of streptococcal sequences from the Ribosomal Database Project (RDP) (Cole *et al.*, 2007). The non-pneumococcal isolates in the study were included to follow up the detection of *cps* genes during blind-test analysis on the serotyping array.

#### PCR of the *cps* region.

All PCRs were performed using the TaKaRa LA PCR kit in 50 µl final volumes sealed with mineral oil, according to the manufacturer’s instructions. Amplification of the complete *cps* region was achieved using primers within the flanking genes *dexB* and *aliA* (Table 2). Where the products were of unknown length, a touchdown PCR program was used, varying the annealing temperature from 68 to 60 °C (decreasing over eight cycles), and then the extension time from 22 to 31 min (increasing over 27 cycles). For cases where the size of the product was known to be 10 kb or under, a simple PCR program using a 60 °C annealing temperature and 10 min, 72 °C extension was used. Products were checked by gel electrophoresis before sequencing.

**Table 2.** Screening primer sequences for groups NT1, NT2 and NT3 See Table S2 for details of use and product sizes.

Primer and application	Sequence (5′–3′)
Cps_F	GACCAAGAATACCGCGAAAA
Cps_R	AACATCCTTCCATTCATCCC
Spans the *cps* locus, primers in conserved *dexB* and *aliA* flanking genes. May be used to identify deletions by length (NT1)	
nspA_F	GATGAGTTTGGCAAGCGTGG
nspA_R	AAGCAAGTGCAACATTGTCC
Within gene *nspA* (NT2)	
aliB1_F	AAAGTGGCTCTTAGGAGCAGG
aliB1_R	TTGCCARTTRTTGAAGGC
Within the first *aliB* gene (NT3 and non-pneumococcal)	
aliB2_F	GATGGTTTGYTWGAAAATGAC
aliB2_R	AGAGARTTRTCAATCATCCAAGC
Within the second *aliB* gene (NT3 and non-pneumococcal)	

Screening primers for genes of interest were generated from the established sequence, and used to assess the content of the remaining sample set where full *cps* PCR was not successful (see Table 2 for primers, and Table S2 for supplementary primers and screening protocols).

#### Sequencing.

For large PCR products, short insert libraries were created (McMurray *et al.*, 1998) and sequenced by capillary. Small PCR products (under 1.5 kb) were end-sequenced by capillary (Sanger *et al.*, 1977).

#### Analysis.

The sequence data were aligned and manipulated in Gap4 (Bonfield *et al.*, 1995), genes were predicted using Glimmer3 (Delcher *et al.*, 1999) and visualized and curated in Artemis (Rutherford *et al.*, 2000), and conserved domains were identified using MotifScan (Hulo *et al.*, 2008). Insertion sequences were identified using the IS Finder database (Siguier *et al.*, 2006). Alignments and trees were constructed using Muscle (Edgar, 2004) and Seaview (Gouy *et al.*, 2010), respectively. Trees were created using the Bio-NJ Jukes–Cantor distance method.

## Results

**Fig. 1.**
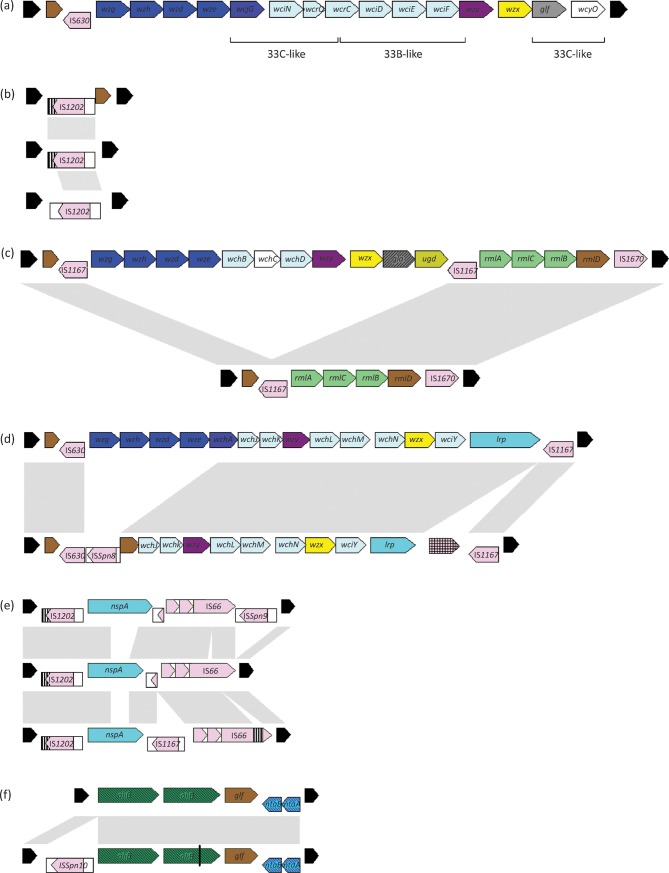
*cps* locus gene content in non-typable and mistyped *S. pneumoniae.* Colour scheme is based on that of Aanensen *et al.* (2007): dark blue (regulatory genes), dark purple (initial transferase), violet (polymerase), yellow (flippase), pale blue (glycosyltransferase), white (acetyltransferase), pink (transposase, insertion sequence delimited by box), hatched pink (group II intron), green (dTDP-l-rhamnose pathway genes), light brown (glucose dehydrogenase), hatched grey (epimerase), grey (UDP-galactopyranose mutase), blue (surface protein), hatched green (*aliB*), hatched blue (toxin–antitoxin), dark brown (pseudogene), black (flanking genes). (a) Isolate 557B. The gene cluster is a mosaic of serotype 33B- and 33C-like genes with divergent regulatory genes, polymerase and flippase. The novel cluster produces a capsular polysaccharide that is serologically type 33C but is predicted to have a different subunit structure. (b) Complete *cps* gene cluster deletions. Isolate 90852, top, retains a truncated *glf* along with an IS*1202*-like sequence that is truncated at the 3′ end by a RUP element (striped box). Isolate 108225, middle, has only the insertion sequence IS*1202*. Isolate 2489-06, bottom, contains the complete IS*1202* sequence. Nucleotide similarity is indicated by grey boxes. (c) Serotype 1, top, and the deletion in isolates 1621, 1629, 1630 and 1636, bottom. The functional portion of the biosynthesis cluster has been lost, presumably due to recombination between the identical flanking IS*1167* sequences. Nucleotide similarity is indicated by grey boxes. (d) Serotype 14, top, and the deletion in isolate 96650, bottom. The regulatory genes are absent, in their place is a novel insertion sequence, IS*Spn8*. The surface protein gene *lrp* is shorter due to a reduced number of repeat domains, and a group II intron has inserted downstream of the cluster. Nucleotide similarity is indicated by grey boxes. (e) Aligned examples of *nspA* clusters. Isolates 890, top; 933, middle; 2437, bottom. Nucleotide similarity is indicated by grey boxes. (f) Comparison of the widespread *aliB*-containing cluster 1, top, and cluster 2, bottom. Cluster 2 is similar to 1, with the addition of the novel IS*Spn10* and a premature stop in the second *aliB*. Nucleotide similarity is indicated by grey boxes.

Sequencing results are shown in Table S1 and divided into five groups: functional *cps* clusters producing a polysaccharide capsule but with genes different to the reference strains (Bentley *et al.*, 2006) (Fig. 1a), complete or partial deletions in the *cps* region rendering the cluster non-functional (group NT1) (Fig. 1b, c), *cps* containing a novel putative surface protein gene (group NT2) (Fig. 1e), *cps* containing a conserved *aliB* gene cluster (group NT3) (Fig. 1f), and non-pneumococcal streptococci with a similar *aliB* gene cluster. These five groups are summarized below.

Three samples were referred from array analysis as they produced a capsule which conflicted serologically with the array prediction. Strain 557B (GenBank accession no. HE651321) is serotype 33C by Quellung reaction, with a mixture of 33B- and 33C-like genes as designated by array. Sequencing of the entire region confirmed that this isolate has a mosaic *cps* cluster of 33B and 33C genes with a divergent *wzx* and *wzy*. Samples 1772-40b and L2008-01622 (accession nos HE651300 and HE651318) are serotype 22F but with two genes absent according to array result. Targeted sequencing of the expected location of these genes showed two novel genes, predicted to serve the same function as those in the reference sequence.

Three non-encapsulated samples (accession nos HE651312, HE651315, HE651319) had no *cps* genes when tested with the array and upon sequencing were found to have had the entire cluster deleted. A further five samples (accession nos HE651299, HE651301, HE651302, HE651303, HE651314) were shown to have undergone partial deletions of the capsule biosynthesis cluster, rendering them non-functional.

In addition to the three isolates with complete *cps* deletions, 12 samples that had no genes represented on the array were sequenced and found to possess a putative novel surface protein gene (*nspA*) at the locus along with a variety of intact and disrupted IS elements (accession nos HE651278–83, HE651285–7, HE651289, HE651297, HE651308). The *nspA* gene itself showed high levels of conservation in some areas but with a hypervariable repeat region: no two isolates were identical.

The galactopyranose mutase gene *glf* was seen in 24 pneumococcal samples (accession nos HE651275–7, HE651284, HE651288, HE651290–6, HE651298, HE651304–7, HE651309–11, HE651313, HE651316–7, HE651320) alongside a conserved gene cluster with *aliB*-like genes, similar to that described elsewhere in non-encapsulated lineages (Hathaway *et al.*, 2004). In 14 cases a putative toxin–antitoxin system was also present (strains marked as ‘cluster 1’ and ‘cluster 2’ in Table S1).

Eleven non-pneumococcal isolates (accession nos HE651264–74) were referred from the array as positive for *glf*. All were shown also to have *aliB* genes very similar to non-encapsulated pneumococci. The *aliB* gene sequences of these and the pneumococci do not cluster according to the species or geographical location of isolation.

## Discussion

### Conflicts between serotyping and microarray

Isolate 557B (Fig. 1a) is serologically type 33C, reacting with antiserum 33e, but has a mosaic *cps* cluster made up of 33B-like and 33C-like genes. *wcjG*, *wciN*, *wcrO* and the first half of *wcrC* are similar to the 33C reference sequence (99 % nucleotide identity), while the second half of *wcrC*, and *wciD*, *wciE* and *wciF*, are similar to 33B (99 % nucleotide identity). The polymerase and flippase *wzy* and *wzx* are not similar to any known serotypes, which is consistent with their function transporting and polymerizing a different subunit structure. Following *wzx* are 33C-like *glf* and *wcyO* (98 % nucleotide identity).

Predicting the structure using the association of function with protein families (Aanensen *et al.*, 2007) suggests that most of the polysaccharide repeat subunit of 557B is 33B-like, with the exception of the 33C-like glycosyltransferase WciN, which may or may not be functional in serotype 33C. As the structure of 33C has not been elucidated, the acetylation pattern brought about by WcyO cannot be inferred. The Wzy-mediated linkage cannot be predicted from the DNA sequence.

The sequence of 557B has greater than 99 % nucleotide identity with partial sequences of an unpublished pneumococcal strain described as a new serogroup 33 member, 33E (gi: 46277554, 158454747, 158454749), including the divergent *wzx* and *wzy* genes. Discriminatory serum development is ongoing: currently this appears similar to antiserum 33e, having a positive reaction to 33C and a weak reaction to 33F.

Isolate L2008-01622 is serologically type 22F but does not have the glycosyl and acetyltransferases *wcwA* and *wcwC* according to array analysis. In their places are divergent genes that contain conserved glycosyltransferase domains and acetyltransferase hexapeptide repeats, respectively. More work is needed to confirm the function of these genes.

Further investigation into the reference strain for 22F (Bentley *et al.*, 2006) led us to discover that the published sequence for this type is not representative of the genes present at its *cps* locus. The genes in place of *wcwA* and *wcwC*, reported here as strain 1772-40b, are identical to those of L2008-01622. These genes have not been reported elsewhere and so may not be present in all 22F isolates; however, they are present in the reference strain.

The presence of serotypable isolates that do not possess the expected *cps* genes demonstrates the diversity that may exist within a serotype and the importance of screening all capsule biosynthesis genes when attempting to infer serotype by DNA-based methods.

### Deletion of genes at the *cps* locus: group NT1

Isolates GM90852, GM108225 and 2489-06 have a complete loss of the *cps* gene cluster, as illustrated in Fig. 1(b). At the locus the first has a putative IS*1202* and truncated *glf*, while the last two show only IS*1202*. The insertion sequence itself is truncated at the 3′ end by a RUP (repeat unit of pneumococcus) element (Oggioni & Claverys, 1999) in GM90852 and GM108225. These latter two isolates represent independent deletion events, as the 300–700 bp of flanking DNA is different in each. Non-typable pneumococci with a complete loss of *cps* genes can be identified by PCR screening, as described in Table S2.

Disruption or partial deletion of the *cps* locus is a common inactivator of capsule biosynthesis, for example making up 13 % of the non-typable isolates in an Australian carriage study (Marsh *et al.*, 2010). Four invasive isolates, L2008-01621, L2008-01629, L2008-01630 and L2008-01636, were found to possess only the rhamnose genes *rmlACBD* by array: the fully sequenced locus also contains flanking IS*1167* elements and an *aliB* pseudogene. The *rmlACBD* cluster is identical to that of serotype 1 (Fig. 1c), suggesting that these isolates lost the functional portion of the capsule cluster by recombination at the identical flanking IS*1167* sequences, similar to the *cps* acquisition scenario described elsewhere (Muñoz *et al.*, 1997). Non-encapsulated pneumococci rarely cause invasive disease. Demonstrating that the deletion existed before disease is beyond the scope of this paper; however, the presence of four identical deletion events from four patients seems unlikely to have been an *in vitro* event after isolation.

A deletion from serotype 14 was also predicted by the array, with genes encoding the capsule subunit relocation machinery and the first 330 bp of the initial transferase all absent from sample GM96650 (Fig. 1d). A novel IS*30* family insertion sequence IS*Spn8* is present at the site of the 3979 bp deletion. The surface protein gene *lrp* is shorter in sample GM96650 (2163 bp) than in other reported sequences {CGSP14 [gi: 182682970], 2499 bp; strain 34359 [gi: 68642995], 4216 bp; JJA [gi: 225722171], 6174 bp}. This is due to the presence of only two copies of the Cna protein B-type repeat (PF05738) compared with five copies in JJA. The capsule gene cluster also contains an inserted, but not disruptive, group II intron.

Lineages which are normally encapsulated have been shown here to have become non-typable through complete or partial deletion of the capsule gene cluster, a division among the non-typables that we designate NT1, similar to the ‘NT group I’ of a recent serotyping paper (Yu *et al.*, 2011). The isolates were from both carriage and disease, suggesting that it may not always be disadvantageous to lose the capsule.

### Putative novel surface protein NspA: group NT2

Twelve isolates (Tables 1 and S1) from Thailand and the Netherlands have no *cps* genes at the locus, but instead contain a gene predicted to produce a novel surface protein, *nspA* (non-typable pneumococcal surface protein). *nspA* is of variable length, ranging from approximately 1.1 to 1.7 kb among these isolates.

Upstream of *nspA* are −10 and −35 promoter sequences. Analysis of the predicted amino acid sequence suggests that there is a cleavable signal peptide, an LPXTG surface anchor motif, and a variable-length glutamic acid-rich helical repeat region from 3 to 27 repeats. Excluding the repeat region, the encoded protein differs by 4 % of the amino acids among all sequenced isolates. Half of these differences are present in only two isolates: RUNMC819 and 07B00830. There is an identical frameshift in four samples (RUNMC819, 07B00830, 08B00930 and 08B01575), confirmed by resequencing, caused by a single base deletion that affects codon 134/135.

The predicted protein contains a conserved KRNYPT motif that may indicate a human polymeric Ig receptor (hpIgR) binding function similar to that of the pneumococcal CbpA (Elm *et al.*, 2004). pIgR is an integral membrane glycoprotein of mucosal epithelial cells, crucial in the release of secretory IgA into the mucosal secretions. The extracellular region has five Ig-like domains, D1–5, of which D3 and D4 have been shown to interact with the YRNYPT motif of CbpA. CbpA–hpIgR binding *in vitro* leads to adhesion to the epithelium and internalization (Elm *et al.*, 2004), and NspA may have a similar function.

*nspA* is flanked by combinations of intact and disrupted IS elements, illustrated in Fig. 1(e). All sequenced samples contain a partial IS*1202* truncated by a RUP element identical to that seen in the *cps* deletions (group NT1), and a putative IS*66*-family sequence with 93 % nucleotide identity to IS*66* element ORF1, 2 and 3 in TIGR4. In sample RUNMC2437, the IS*66*-like sequence contains a RUP element that disrups ORF3, at the same site as in TIGR4 and in published encapsulated sequences such as serotype 43.

Four samples also contain an intact IS*1167*, similar at 93 % nucleotide identity to TIGR4 IS*1167*, while in eight other samples only the 3′ end of IS*1167* is present. As well as IS*1202* and IS*66*, sample 07B00890 contains a novel IS*30*-family insertion sequence, IS*Spn9*, most similar to *Streptococcus mitis* B6 IS*Smi3* (94 % nucleotide identity).

There are a variety of insertion sequences flanking *nspA*, some of which are found in typable *cps* loci and may therefore provide potential recombination points, facilitating the spread of the gene between pneumococcal lineages. It is consistent with the observation that the MLST data for these strains (Table S1) show clearly that *nspA* is not restricted to a single lineage of closely related pneumococci, but is instead found in isolates that appear to be distantly related. This, taken together with the source of these strains from locations as distant as Thailand and the Netherlands, also indicates that strains bearing this gene are quite successful.

Authors’ note: after the acceptance of this manuscript, the nucleotide sequences of other examples of this gene were released, named *pspK.* To our knowledge, *pspK* has not yet been described, but it leads us to conclude that the recent grouping ‘NT group II clade I’ (Yu *et al.*, 2011) is similar to NT2.

### *aliB-*like genes in *S. pneumoniae*: group NT3

Twenty-four samples have a well-conserved *aliB* gene cluster (Fig. 1f). The non-encapsulated sequence types 448 and 449 are among these, lineages which circulate internationally, and can make up 11 % of non-typable isolates in carriage (Marsh *et al.*, 2010) and have been associated with conjunctivitis in the USA for more than 20 years (Hanage *et al.*, 2006). For 14 samples (‘cluster 1’ and ‘cluster 2’; Table 1) the locus is almost identical to that described in strain 110.58 (Hathaway *et al.*, 2004), comprising two non-identical *aliB* genes, a *glf* pseudogene and a putative toxin–antitoxin system. A further two pneumococcal isolates have only one *aliB.* AliB has been shown to aid colonization in two knockout studies (Hathaway *et al.*, 2010; Kerr *et al.*, 2004), affecting uptake of glutamic acid and early growth rates in a mouse model.

The sequence of cluster 1 is identical to published strain 110.58 [gi: 50540968]. Cluster 2 is similar, but the genes are preceded by a novel IS*110*-family insertion sequence, IS*Spn10*, and the second *aliB* gene is disrupted by a premature stop codon in all cases.

**Fig. 2.**
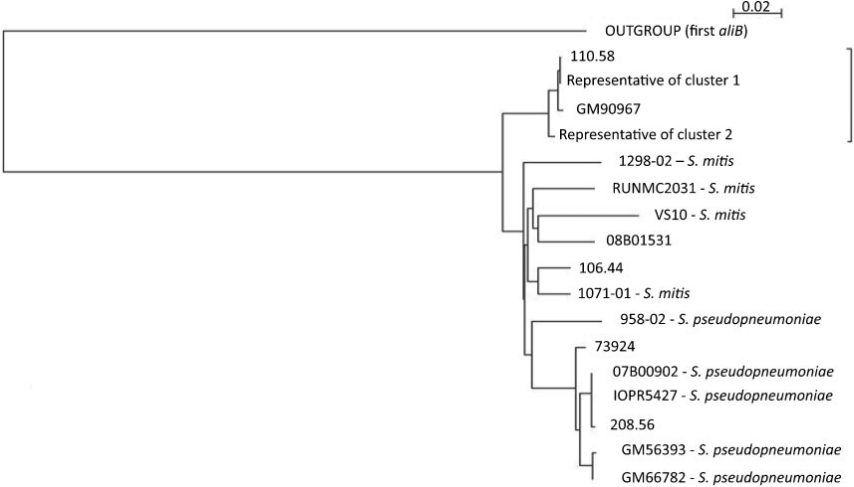
Tree of nucleotide similarity of the second *aliB* gene. A single representative from the identical *aliB* cluster 1 and 2 was used along with divergent pneumococcal sequences, non-pneumococcal streptococci and three published pneumococcal strains: 110.58, 106.44 and 208.56 (Hathaway *et al.*, 2004). The outgroup is an aligned sequence from the first *aliB* in the cluster of strain 110.58. Where a species is not specified, the isolate is *S. pneumoniae*. Although *S. mitis* and *S. pseudopneumoniae* tend to fall together, pneumococcal isolates are also found in those groups, suggesting that these genes have been acquired by different species recently. The branch identified by the bracket contains isolates that also have the toxin–antitoxin system at the *cps* locus: the *aliB* sequence is highly conserved and geographically widespread.

The putative toxin–antitoxin system of cluster 1 and cluster 2, *ntaA* and *ntaB* (non-typable toxin-antitoxin gene A, antitoxin, and B, toxin), is similar to two *Lactobacillus salivarius* UCC118 genes [gi: 90821554, gi: 90821553] and their flanking sequences with 87 % nucleotide identity. *ntaB* contains a conserved Fic/DOC domain. The presence of a maintenance system such as this may contribute to the persistence of this gene cluster in temporally and geographically distant isolates, and provide some explanation for the divergence of the identical *aliB* sequences in this clade compared with pneumococcal and non-pneumococcal *aliB* clusters that lack *ntaAB* (Fig. 2).

### Other streptococci

Eleven non-pneumococcal carriage isolates with *glf* genes identified by microarray also contain *aliB* genes. Six *Streptococcus pseudopneumoniae,* three *S. mitis* and one unidentified isolate have a single *aliB*, while one *S. mitis* contains two *aliB* genes (Table S1). Where a single gene is present, it is most similar to the second consecutive *aliB* of the group NT3 clusters. All of these *aliB*s are more similar to non-typable *S. pneumoniae* (97 and 94 % nucleotide identity for the first and second *aliB*, respectively) than published *S. mitis* {[gi: 92109246] 81 and 79 %, respectively} or *Streptococcus oralis* sequences {[gi: 92109243] 81 and 80 %, respectively}.

As shown in Fig. 2, the sequences of *aliB* do not cluster exclusively by species or geographical origin, in keeping with the known presence of inter-species recombination between nasopharyngeal streptococci (Donati *et al.*, 2010). Isolates such as 0900-07 also fall into different groups when classified by the sequence of the first or second *aliB* (data not shown), suggesting that there may be a mosaic acquisition of these genes. Non-pneumococcal streptococci have capsule-like genes at the *cps* locus, such as the RPS (receptor polysaccharide) cluster in *S. oralis* (Yang *et al.*, 2009) and *S. mitis* (Yoshida *et al.*, 2006), and they have been shown to be functionally transferrable between species (Yang *et al.*, 2009). The occurrence of *aliB* genes among several commensal species is further evidence that streptococci have a large reservoir of genetic material at their disposal.

### Conclusion

The results described here reveal previously unknown variation at the *cps* locus. As well as divergent genes directly involved in capsule synthesis, we have found others such as *aliB* and *nspA* that may be advantageous in carriage, and instances of capsule inactivation by deletion.

The mosaic acquisition of capsule biosynthesis genes from serotype 33B and 33C clusters in 557B demonstrates the potential for novel pneumococcal serotypes to be generated by recombination. Conversely, two 22F strains possess novel glycosyl and acetyltransferases that differ from the reference sequence, indicating that caution is required when DNA-based serotyping is reliant on few sequenced isolates.

Non-pneumococcal streptococci such as *S. mitis* can have capsule-like genes at an equivalent locus to *S. pneumoniae.* Pathogen and non-pathogen species are known to recombine with one another, and the ultimate origins and evolutionary history of the capsule loci may include recombination between the pneumococcus and other streptococcal species. Here we have shown that *S. pneumoniae*-like *aliB* genes are present in other species, do not cluster according to species or geographical provenance, and so may be circulating globally in the nasopharyngeal microbiota genetic pool.

The highly recombinogenic capsule locus is a straightforward PCR screening target because it is flanked by conserved *dexB* and *aliA* genes. Several novel genes and gene variants are described here with screening primers and expected results to facilitate others in exploring diversity at the *cps* locus in non-typable pneumococci.
